# Cerebral Air Embolism After Pigtail Catheter Drainage for Pneumothorax: A Case Report and Review of the Literature

**DOI:** 10.3389/fsurg.2021.743051

**Published:** 2021-09-28

**Authors:** Yi Chen, Chunhui Zheng, Qinghui Zeng, Fangbiao Zhang, Shaosong Tu, Zhijun Wu

**Affiliations:** ^1^Department of Operating Room, Zhejiang University, Lishui Hospital, Lishui Municipal Central Hospital, Lishui, China; ^2^Department of Cardiothoracic Surgery, Zhejiang University, Lishui Hospital, Lishui Municipal Central Hospital, Lishui, China

**Keywords:** cerebral air embolism, pigtail catheter drainage, pneumothorax, complication, thoracic

## Abstract

**Objective:** Cerebral air embolism (CAE) is an extremely rare but serious complication of pigtail catheter drainage. The aim of the case report is to review our experience in the diagnosis and treatment for CAE after pigtail catheter drainage.

**Case presentation:** In our study, we report a case of CAE following pigtail catheter insertion for pneumothorax. A 50-year-old man was diagnosed with a pulmonary mass in the right lower lobe. He underwent a right lower lobectomy. Pneumothorax was present after the removal of the chest tube. Pigtail catheter drainage was used in order to treat the pneumothorax, which resulted in convulsions, limb stiffness, and unconsciousness. A brain CT scan was immediately performed and showed multiple low densities in the right occipital lobe, which was diagnosed as CAE. Assisted breathing, antibiotic treatment, and antiepileptic therapy were used and the patient gradually improved and was discharged at 27 days of treatment but the muscle strength of the left limb was weakened.

**Conclusion:** We analyzed and summarized the possible causes of CAE in the literature, and the findings of the case could enhance the vigilance of clinicians.

## Introduction

Pneumothorax and pleural fluid are common complications of thoracic surgery. Pigtail catheter drainage is a common and effective treatment (1). Compared with large bore chest tube drainage, the procedure of pigtail catheter drainage is easier to perform and less traumatic (2, 3). Bleeding, infection, chest pain, and re-expansion pulmonary edema are the most common complications of pigtail catheter drainage similar to large bore chest tube drainage (1).

Cerebral air embolism (CAE) is an extremely rare but serious complication that has not been paid enough attention to in the medical invasive process such as percutaneous transthoracic lung biopsy (4), pigtail catheter drainage (1), and thoracic surgery (5). In this article, we report a case of a serious complication of CAE after pigtail catheter drainage and review the published literature.

## Case Presentation

**Figure 1:**
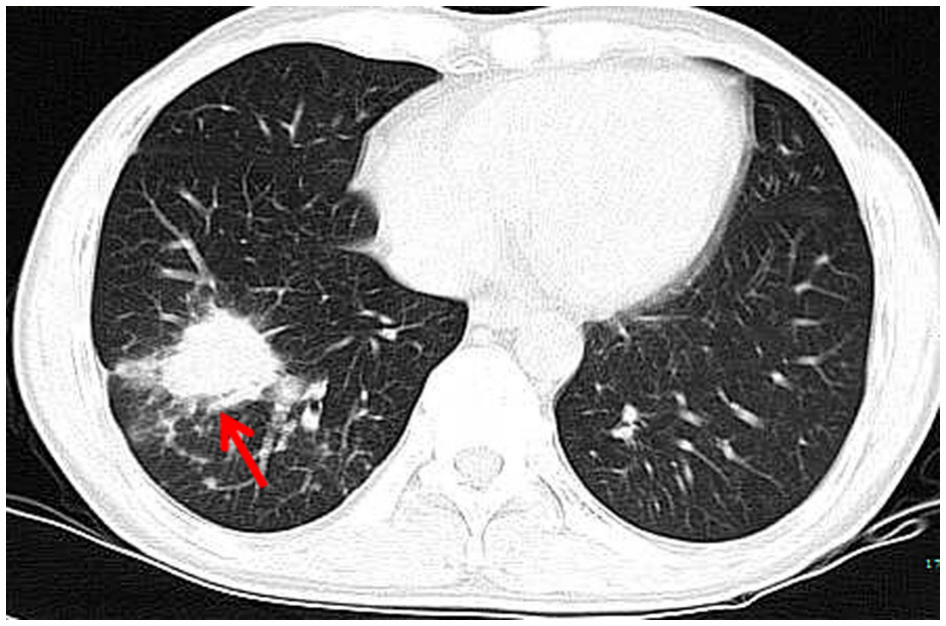
A chest CT scan revealing a mass measuring 3.7 × 3.0 cm in size in the right lower lung (red arrow).

**Figure 2:**
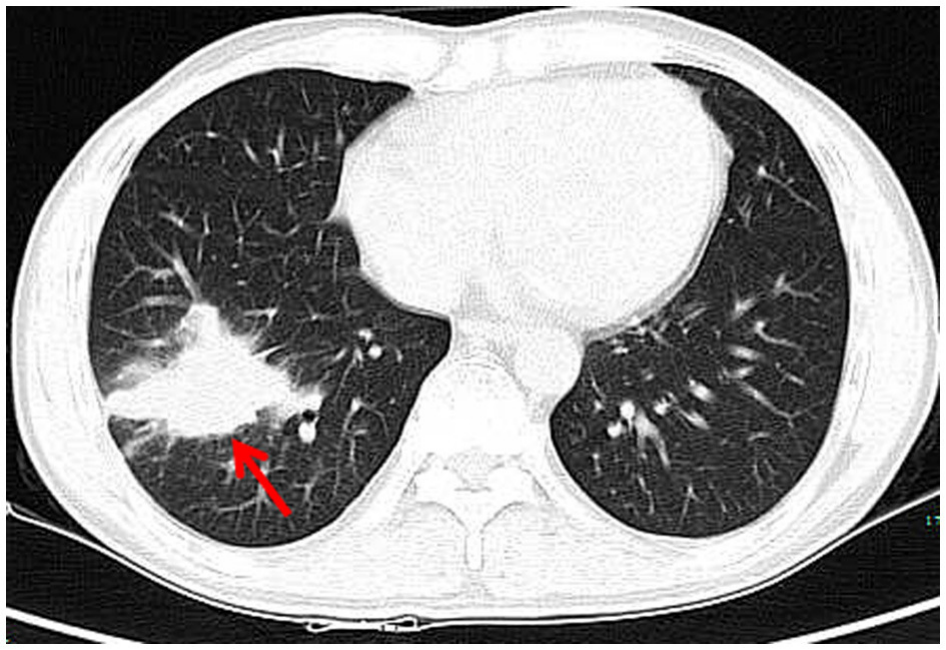
Chest CT showed that the right lower lung mass had not shrunk after anti-infective treatment (red arrow).

**Figure 3:**
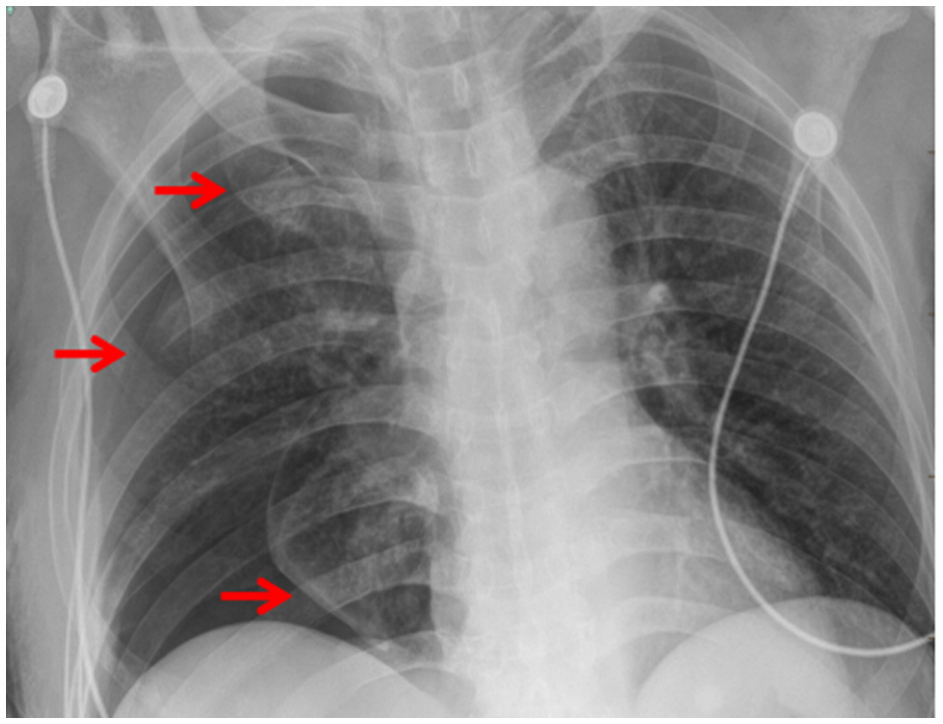
Pneumothorax was presented after the removal of the chest tube in x-ray (red arrow).

**Figure 4:**
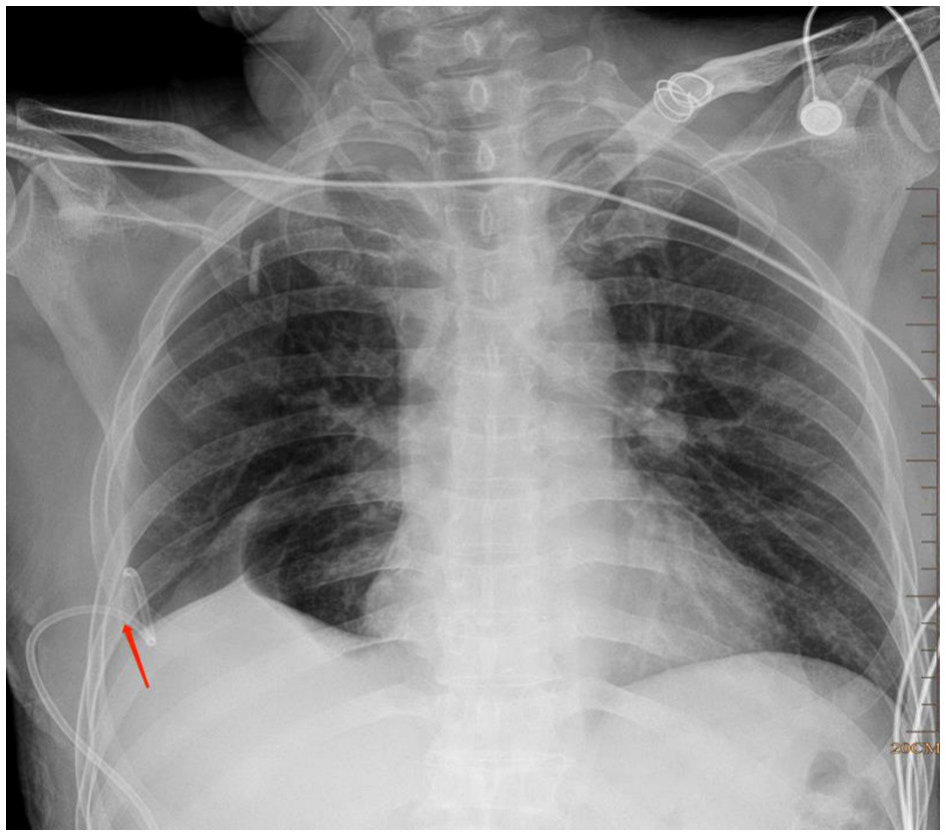
A pigtail catheter was inserted into chest for pneumothorax (red arrow).

**Figure 5:**
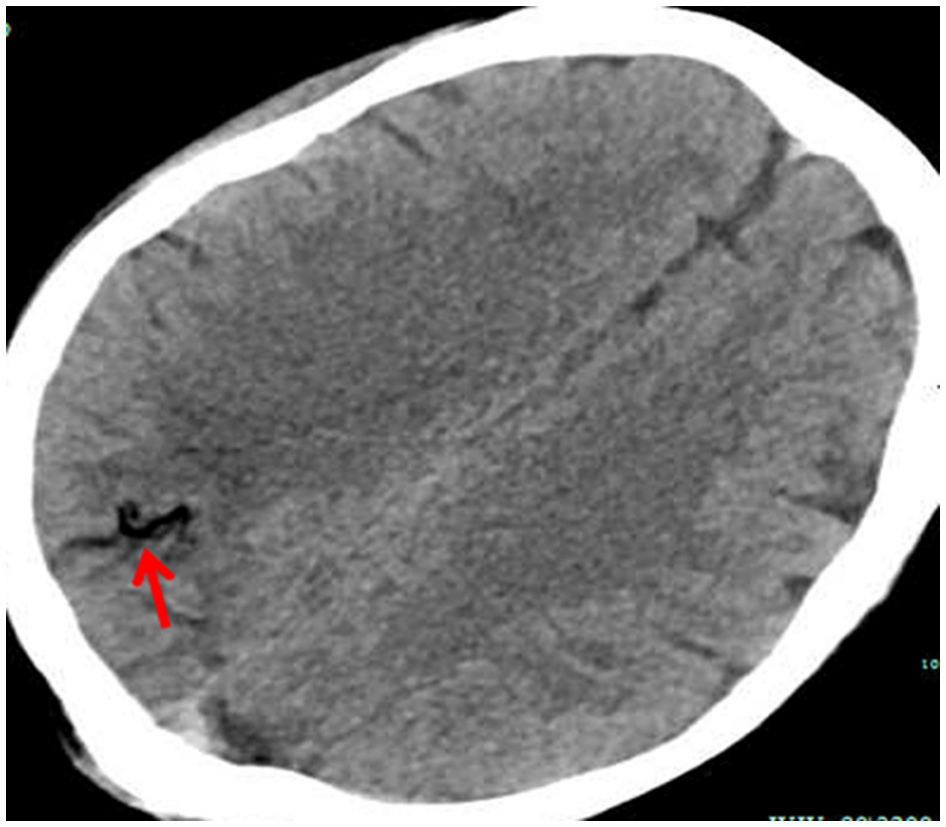
A brain CT scan showed multiple low densities in the right occipital lobe (red arrow).

A 50-year-old man was presented to our hospital (Lishui Municipal Central Hospital, Lishui, China) with cough and sputum that lasted for 2 months and blood-stained sputum that lasted for 1 month. The patient had a history of type II diabetes mellitus and cigarette smoking and no history of hypertension, hepatitis, tuberculosis, or coronary heart disease. The laboratory test results were white blood cell count 9,900 cells/ml (normal range, 4,000–9,500 cells/ml); hemoglobin 144 g/l (normal range, 130–175 g/l); platelets 3,160 cells/ml (normal range, 1,250–3,500 cells/ml) and C-reactive protein 15.6 mg/dl (<8 mg/dl). A chest CT scan (Brilliance iCT; Philips Healthcare, Amsterdam, The Netherlands) revealed a mass in the right lower lung (Figure 1). Based on the history and imaging examination, lung abscess was considered. Antibiotic treatment was used for 2 months. Unfortunately, the mass did not shrink (Figure 2). Antibiotic treatment failed to improve the right pneumonic infiltration. A right lower lobectomy was performed under general anesthesia on August 18, 2020. The chest tube was removed on postoperative day 4. Unfortunately, pneumothorax was presented after the removal of the chest tube (Figure 3). So, a pigtail catheter was inserted for pneumothorax (Figure 4). The pigtail catheter was placed through a guide wire after thoracentesis (1). As soon as the guide wire was inserted, he presented convulsions, limb stiffness, and unconsciousness. A brain CT scan was performed immediately and showed multiple low densities in the right occipital lobe (Figure 5), which were diagnosed as CAE. Assisted breathing, antibiotic treatment, and antiepileptic therapy were used and the patient gradually improved and was discharged at 27 days of treatment but the muscle strength of the left limb was weakened. At 3 months follow-up, the left muscle strength of the patient had not recovered.

## Discussion

Chest tube and pigtail catheter drainage are the main methods for the treatment of pneumothorax and pleural effusion. Hemothorax, pneumothorax, pain, and infection are common complications (1). Because of the less tissue injury and limitation of the movement of patients, the application of pigtail catheter drainage is more and more in a clinic. Furthermore, pigtail catheter drainage was shown to be as effective as chest tube drainage in the treatment of various chest diseases (1).

Cerebral air embolism is a rare but serious complication of pigtail catheter drainage, which can cause cerebral ischemia and hypoxia brain (6). Some patients recover smoothly without any neurologic complications (1), while a small number of patients have residual nerve injury symptoms (5) or even death (7). To the best of our knowledge, CAE occurring after the pigtail catheter insertion for drainage because of pleural effusion, which was initially reported by Kim SI in 2013 (1). However, CAE occurring after the pigtail catheter drainage for pneumothorax has not been reported.

The mechanism of CAE caused by pigtail catheter drainage is still unclear. Based on previously published reports, there are several possibilities for the occurrence of CAE. First, it is possible to puncture the lung vein. Air enters the pulmonary venous system when the intrathoracic pressure exceeds the pulmonary venous pressure (1). Second, gas is injected unintentionally into a vein in the chest wall or pleura when an anesthetic is injected, causing a CAE (1). Third, the air entering the pulmonary venous system exceeded the filtration capacity of the lungs, causing the air from the venous circulation to enter the arterial circulation and eventually form CAE (5, 8, 9). Fourth, when the gas enters the arterial circulation through an intrapulmonary arteriovenous fistula or cardiac shunt pathway (5). In our case, however, preoperative contrast-enhanced CT showed no intrapulmonary arteriovenous fistula, and cardiac color ultrasound showed no intracardiac defect, such as atrial septal defect and patent foramen ovale, so the cardiac shunt pathway was excluded. For the case in this report, it is most possible that air enters the brain because the puncture needle penetrated the pulmonary vein under tension pneumothorax.

The clinical symptoms of CAE largely depended on the quantity of gas and the areas of the brain that are affected (5). When the quantity of gas is less, patients may present with no clear clinical symptoms. As the gas increases, patients present with different symptoms, such as headache, hemiplegia, convulsion, loss of consciousness, and coma (6). In our case, the patient immediately performed convulsions, limb stiffness, and unconsciousness due to the large quantity of gas in the brain. According to sudden changes in clinical manifestations, neurological disorders should be considered. However, specific causes such as thromboembolism, cerebral hemorrhage, or gas embolism still need further examination. Early recognition is the best treatment strategy for CAE. Cerebral CT is considered to be a rapid, definitive, and effective tool of examination. The most characteristic appearance is the presence of air bubbles in the cerebral arteries (1).

High-flow oxygen therapy reduces emboli volume and maximizes tissue oxygenation (10), which is an effective treatment for CAE. However, the most effective treatment for CAE is hyperbaric oxygen therapy (HBOT). This therapy increases the oxygen solubility, reduces the volume of air emboli, reduces vascular endothelial damage, decreases the permeability of blood-brain barrier, and reduces cerebral edema (1, 7). In our case, the patient received high-flow oxygen therapy and did not receive HBOT because of pneumothorax.

How to avoid such complications is worth learning for every thoracic surgeon. The authors think that chest CT or x-ray should be carefully read during the process of puncture. Second, the needle should not go too deep into the chest to avoid lung damage. Finally, the needle should be stabilized after entering the chest to avoid unnecessary injury caused by needle activity.

## Conclusion

In this article, we report a rare case of a serious complication of CAE after pigtail catheter drainage for pneumothorax. There are three important findings. First, the occurrence of CAE by pigtail catheter drainage is rare but serious and potentially fatal, which should be paid enough attention to in the thoracic medical process. Second, the puncture needle and guidewire are not to be inserted too deep as it is possible to puncture the lung vein. Third, high-flow oxygen therapy, HBOT, and cardiopulmonary supportive care are thought to be the effective treatment for CAE.

## Data Availability Statement

The original contributions presented in the study are included in the article/supplementary material, further inquiries can be directed to the corresponding author/s.

## Ethics Statement

Written informed consent was obtained from the individual(s) for the publication of any potentially identifiable images or data included in this article.

## Author Contributions

YC and ZW drafted the manuscript. ST, ZW, QZ, and FZ performed the surgery. CZ helped collect clinical data and made critical revisions for important intellectual content. All the authors read and approved the final manuscript.

## Conflict of Interest

The authors declare that the research was conducted in the absence of any commercial or financial relationships that could be construed as a potential conflict of interest.

## Publisher's Note

All claims expressed in this article are solely those of the authors and do not necessarily represent those of their affiliated organizations, or those of the publisher, the editors and the reviewers. Any product that may be evaluated in this article, or claim that may be made by its manufacturer, is not guaranteed or endorsed by the publisher.

## Abbreviations

CT: Computed tomography
CAE: Cerebral air embolism
PTLB: Percutaneous transthoracic lung biopsy
WBC: White blood cell
HBOT: Hyperbaric oxygen therapy.
